# An Explainable Framework for Mental Health Monitoring Using Lightweight and Privacy-Preserving Federated Facial Emotion Recognition

**DOI:** 10.3390/s25237320

**Published:** 2025-12-02

**Authors:** Dina Shehada, Hissam Tawfik, Ahmed Bouridane, Abir Hussain

**Affiliations:** 1 Department of Electrical Engineering, University of Sharjah, Sharjah P.O. Box 27272, United Arab Emirates; htawfik@sharjah.ac.ae (H.T.); abir.hussain@sharjah.ac.ae (A.H.); 2 Department of Engineering and Information Technology, University of Dubai, Dubai P.O. Box 14143, United Arab Emirates; 3 School of Built Environment, Engineering and Computing, Leeds Beckett University, Leeds LS1 3HE, UK; 4 College of Computing and Informatics, University of Sharjah, Sharjah P.O. Box 27272, United Arab Emirates; abouridane@sharjah.ac.ae

**Keywords:** explainable AI (XAI), facial emotion recognition (FER), trustworthy AI, model interpretability, assistive technology, federated learning

## Abstract

The continuous analysis of emotional cues through facial emotion recognition (FER) systems can support mental health evaluation and psychological well-being monitoring systems. Most FER systems face privacy and trust concerns due to their centralized data approaches and lack of transparency, making potential deployment difficult. To address these concerns, a federated, explainability-driven FER framework designed to provide trustworthy and privacy-preserving emotion recognition with potential applications in mental health monitoring is proposed in this paper. The proposed lightweight Convolutional Neural Network (CNN) enables real-time inference while preserving high accuracy. Comprehensive evaluations on RAF-DB, ExpW, and FER2013 datasets, show that the proposed model demonstrates improved cross-dataset generalization compared to related works, achieving average accuracies of 75.5% and 74.3% in centralized and federated settings, respectively. Quantitative perturbation-based metrics, including Insertion and Deletion Area Under Curve (IAUC and DAUC), Average Drop (AD), Increase in Confidence (IC), Average Drop in Accuracy (ADA), and Active Pixel Ratio, were employed to objectively evaluate the quality and reliability of the model Grad-CAM++ explanations. The results confirm that model explainability enhances transparency and is directly associated with improved model performance.

## 1. Introduction

Mental health plays a critical role in overall human well-being. However, millions of individuals worldwide remain undiagnosed or untreated due to limited access to care, social stigma, and shortages of trained professionals. The World Health Organization (WHO) [1] estimates that nearly one billion people suffer from mental illnesses like anxiety and depression, which continue to impose increasing social and economic burdens. In order to tackle these issues, the WHO Comprehensive Mental Health Action Plan 2013–2030 [2] emphasizes technology-enabled support, community-based intervention, and early detection. Early detection and ongoing monitoring of emotional states could support prompt intervention and individualized support. Advances in artificial intelligence (AI) have enabled emotion recognition from multiple modalities such as facial expressions, speech, and physiological signals. Among these, facial emotion recognition (FER) has become a popular, non-invasive, and informative technique for determining emotional states, which makes it suitable for assistive technology with potential applications in mental health monitoring. Psychological studies have shown that emotional balance strongly correlates with psychological resilience and overall well-being. Fredrickson and Losada’s work on the positivity ratio underscores that individuals who maintain a higher frequency of positive emotions tend to experience improved mental health outcomes [3,4]. These findings highlight the importance of reliable and continuous emotion assessment tools in supporting mental health care.

The deployment of facial emotion recognition (FER) in mental health applications remains constrained by two major challenges: privacy and interpretability. State-of-the-art FER systems rely on centralized data storage, which raises substantial privacy concerns when managing sensitive personal and clinical information. Additionally, deep learning models often function as “black boxes,” providing no transparency into their decision-making processes or the reliability of their predictions. In healthcare contexts, where accountability, explainability, and trust are essential, the lack of interpretability poses a major barrier to adoption [5].

Federated learning (FL) offers an effective solution to the privacy issue by enabling decentralized training across multiple devices without transferring raw data to a central server [6]. While FL enhances data confidentiality, it inherits the same interpretability challenges as conventional deep models [7]. Explainable artificial intelligence (XAI) methods can bridge this gap by revealing the underlying reasoning behind model predictions and highlighting salient features contributing to emotion recognition. This paper introduces a lightweight, federated learning framework designed for potential edge deployment and enhanced with quantitative explainability assessment for trustworthy and privacy-preserving facial emotion recognition. The approach employs multiple perturbation-based explainability metrics to objectively evaluate explanation quality and model reliability, such as Insertion and Deletion Area Under Curve (IAUC and DAUC), Average Drop (AD), Increase in Confidence (IC), Average Drop in Accuracy (ADA), and Active Pixel Ratio to analyze the relationship between highlighted facial regions and model performance. The lightweight design ensures computational efficiency and suitability for real-time emotion monitoring applications on edge devices, such as assistive or mental health support systems. Embedding interpretability into the optimization and evaluation pipeline promotes transparent and trustworthy decision-making, aligning the framework with ethical standards for mental health–related applications. The main contributions of this work can be summarized as follows:

1. Lightweight and Edge-Deployable FER Architecture: A compact CNN architecture optimized for efficient training and low-latency inference, achieving a strong balance between accuracy and computational cost. The model demonstrates superior cross-dataset generalization across Facial Emotion Recognition 2013 (FER2013), Real-world Affective Faces Database (RAF-DB), and Expression in the Wild (ExpW), reaching 75.5% and 74.3% average accuracy under centralized and federated configurations, respectively. With only 1.45 M parameters and 0.107 GFLOPs, it demonstrates stronger cross-dataset generalization than previously reported cross-dataset FER architectures across all three datasets, while remaining suitable for real-time emotion monitoring on resource-constrained edge devices.
2. Federated Learning with Integrated Explainability for Trustworthy Model Selection: A privacy-preserving federated learning framework that explicitly incorporates explainability into the evaluation and model optimization process. The framework combines federated training with multi-level interpretability analysis, enabling systematic comparison of model behavior across different configurations. By leveraging explainability to guide model selection, the approach enhances transparency, interpretability, and accountability, supporting its suitability for deployment in privacy-sensitive mental health monitoring systems.
3. Quantitative Explainability Assessment: A systematic evaluation of Gradient-weighted Class Activation Mapping++ (Grad-CAM++) explanations using perturbation metrics, including Insertion Area Under Curve (IAUC), Deletion Area Under Curve (DAUC), Average Drop (AD), Increase in Confidence (IC), Average Drop in Accuracy (ADA), and Active Pixel Ratio, to provide objective, reproducible, and comparable measures of explanation quality and model trustworthiness.

The remainder of this paper is organized as follows: Section 2 reviews related works. Section 3 details the proposed framework, including architecture design and metric formulation. Section 4 presents experimental results and explainability analysis. Section 5 presents cross-dataset evaluation and comparative analysis to assess generalization performance. Section 6 examines edge deployment and runtime performance. Section 7 discusses ethical and practical considerations for deploying FER systems in mental health contexts. Finally, Section 8 concludes the paper and outlines future research directions.

## 2. Related Work

Facial Expression Recognition (FER) has gained substantial attention due to its applications across healthcare, human–computer interaction, education, and emotional computing. This section reviews key developments in FER systems, including traditional deep learning-based methods, application-specific frameworks, and recent federated learning approaches for privacy-preserving FER.

### 2.1. FER Systems and Lightweight Models

Minaee et al. [8] proposed an attention-based CNN framework that focused on critical facial regions such as the eyes and mouth using Spatial Transformer Networks. The model achieved accuracies of 70.02% on FER2013, 98.0% on CK+, 99.3% on FERG, and 92.8% on JAFFE. However, since training and testing were conducted separately on each dataset, the model exhibited limited generalization across domains.

Hou et al. [9] introduced a FER system for assessing student engagement, combining Multi-Task Cascaded Convolution Neural Networks (MTCNN) for face detection with a dual-feature extractor using VGG16 and ECANet. The system achieved 67.4% on FER2013 and 99.18% on CK+, whereas Shi et al. [10] attained 71.5% on FER2013 and 98.48% on CK+ with an Multiple Branch Cross-Connected Convolution Neural Network (MBCC-CNN). Despite high accuracy, both approaches were computationally heavy, limiting their deployment on low-resource devices.

To enhance efficiency, Zhou et al. [11] developed a lightweight DenseNet model utilizing Histogram of Oriented Gradients (HOG) for feature extraction, reaching 67% accuracy on FER2013. Similarly, Kim et al. [12] applied Log-Level Threshold Quantization (LLTQ) to reduce computation, achieving 86.5% accuracy on FER+, though energy consumption remained high [13].

Dual CNN models proposed by Saurav et al. [14] reached 72.77% on FER2013 and 98.54% on CK+, while the ensemble-based EmNet [15] achieved 74.1% on FER2013, 84% on RAF-DB, and 53% on SFEW, albeit with large model size (4.81 M parameters). Zhao et al. [16] introduced a lightweight emotion recognition (LER) system emphasizing model compression, achieving 71.55% on FER2013 and 85.68% on FER+. However, their enhanced FERFIN dataset was not publicly released, limiting reproducibility.

Huang et al. [17] combined CNN and squeeze-and-excitation residual networks (SE-ResNet), achieving 83.37% accuracy on RAF-DB dataset after fine-tuning on AffectNet. Dias et al. [18] conducted cross-dataset training using VGG-Face with a Random Patches strategy, attaining 83.6% mean accuracy across diverse datasets, though the study highlighted dataset bias and posed-expression limitations. Liang et al. [19] introduced MSAU-Net for fine-grained FER, focusing on discriminative facial regions and achieving up to 78.3% on FG-Emotions dataset, yet at the cost of high computational demand.

Recent works also explored ensemble and real-time FER. Reghunathan et al. [20] compared transfer learning strategies across ResNet50, InceptionV3, and AlexNet, obtaining 73.56% accuracy on FER2013 but with high computational complexity. Gupta et al. [21] developed a learner engagement detection system using ResNet-50, achieving 73.4% on FER2013 and 89.56% on CK+. Despite good performance, these models still face challenges with real-world generalization, efficiency, and dataset diversity.

Recent studies have explored modeling emotion through richer temporal structures and physiological signals. Hu et al. [22] proposed STRFLNet, an electroencephalography (EEG) based emotion recognition model that captures both stable and rapidly changing patterns in brain activity, leading to clearer and more reliable emotional estimates. Similarly, Cai et al. [23] convert EEG signals into spectral images to learn frequency, and region-related patterns that improve recognition accuracy. These works show that using temporal brain dynamics and physiological information can significantly enhance the robustness and generalization of emotion recognition systems.

### 2.2. FER for Assistive, Clinical, and Contextual Applications

FER has been increasingly applied in assistive and healthcare domains to support mental health assessment and inclusivity. Ma et al. [24] developed a depression detection framework using 14 facial regions of interest (ROIs), Discrete Wavelet Transform (DWT) for optical flow features, and Bayesian Networks (BNs), achieving 81.7% accuracy despite limited data and disconnected BN subnetworks.

Similarly, Shangguan et al. [25] employed a dual-stream Multiple Instance Learning (MIL) framework for depression detection from facial videos, achieving 74.7% accuracy on 150 samples. The system identified depression-indicative frames using attention mechanisms but faced poor generalization due to limited data. Gue et al. [26] retrained EfficientNet for binary depression classification using emotion groupings but reached only 59% accuracy, constrained by subjective emotion mapping and dataset size.

Ramis et al. [27] examined FER for individuals with intellectual disabilities using AlexNet and VGG19 across five datasets, achieving 86–89% accuracy on typical populations but dropping to 18–46% on MuDERI, a dataset of people with intellectual disabilities. Using LIME (Local Interpretable Model-agnostic Explanations) and RISE (Randomized Input Sampling for Explanation), they revealed models’ inability to capture subtle expressive variations, emphasizing inclusivity challenges in current FER systems.

Rathod et al. [28] addressed children’s FER for online learning, introducing a new dataset of 53 videos from 12 children and achieving 89.31% accuracy with ResNet152V2. ScoreGrad provided the most interpretable visualizations among XAI methods, however, it should be noted that their small utilized dataset limited the network generalization. Ruangdit et al. [29] proposed a multimodal FER system combining facial and speech modalities, achieving 85.8% accuracy for facial recognition. Despite promising results, interpretability and clinical validation were lacking.

Hettiarachchi et al. [30] studied gender effects in multimodal FER, finding that gender-specific models trained separately for males and females outperformed gender-neutral ones, particularly in speech emotion recognition.

These studies highlight the importance of inclusivity, personalization, and interpretability in FER for real-world and healthcare applications.

### 2.3. Research Gap

Despite significant advances in FER, the integration of FL with rigorous quantitative XAI evaluation for mental-health-oriented FER remains largely unexplored. Several additional studies have explored explainable FER systems [31,32,33,34,35,36,37,38,39,40,41].

Three critical gaps still limit the clinical deployment of FER systems for mental health monitoring, as outlined below:

- Limited Explainability and Lack of Standardized Quantitative XAI Evaluation: Many mental-health-focused FER studies either ignore explainability [24,25,26] or use it only as a basic visualization tool, limiting the trust and interpretability of their decisions. Even in cases where XAI was used, explanation reliability is primarily evaluated by subjective visual inspection rather than using standardized quantitative metrics [27,28,31,32,33,34,35,37,38].
- High Computational Overhead and Lack of Lightweight Architecture Optimization: Most FER systems use computationally expensive deep learning models unsuitable for resource-constrained environments [26,27,28,29,34,35,36,37,38,40,42,43,44]. Current research lacks lightweight architectures that simultaneously achieve high accuracy and reliable XAI interpretability.
- Limited Cross-Generalization and Privacy-Preserving Deployment: FER systems still struggle to generalize to real-world clinical environments, as many studies rely on a single dataset or controlled, posed emotion data that fail to capture in-the-wild variability [31,32,33,34,39,41]. Federated learning settings also remain limited, often involving few clients and acted datasets, which weakens real-world generalization and deployability [43,45,46]. Additionally, some approaches remain conceptual or compromise privacy, without achieving true cross-dataset learning under privacy-preserving constraints [47].

In order to overcome these constraints, this work presents a lightweight FER framework with thorough quantitative XAI evaluation, providing computationally efficient, interpretable, and privacy-preserving emotion recognition for real-world mental health monitoring applications. Detailed methodology is presented in the following section.

## 3. Proposed Methodology

### 3.1. FER for Mental Health

We formulate the task of emotion recognition for monitoring emotional states with potential support for mental health applications. Emotions such as anger, happiness, sadness, and neutrality are widely recognized as relevant indicators of mood patterns and potential psychological disorders. Accordingly, our study restricts the label space to these four clinically relevant emotions. This selection is supported by their established significance within the domain of mental health [48,49]. Happiness, or more specifically its absence, is a core feature of both depression and anxiety, reflecting impairments in responses to pleasurable experiences. Sadness is a hallmark of depressive disorders and is strongly associated with elevated suicide risk [50,51]. Additionally, various studies have consistently linked anger to depression, emphasizing its role in the onset and persistence of symptoms [52,53]. The inclusion of a neutral state provides an essential baseline for distinguishing affective responses. In particular, the contrast between happiness and neutral is crucial for identifying anhedonia, where emotional experiences are flattened toward neutrality. Our choice is further reinforced by prior FER for mental health studies that employed the same or closely aligned four emotion subset in related monitoring tasks [29,35]. This emphasis on capturing both positive and negative emotions aligns with the positivity ratio, which indicates that psychological well-being is promoted when the frequency of positive emotions is at least three times that of negative ones [3]. Validation on clinically annotated depression datasets would be ideal; however, such datasets are currently unavailable publicly due to privacy and ethical constraints. Our evaluation demonstrates the technical feasibility of the framework using established emotion categories with documented clinical relevance.

### 3.2. Federated Learning Collaborative Training

**Figure 1:**
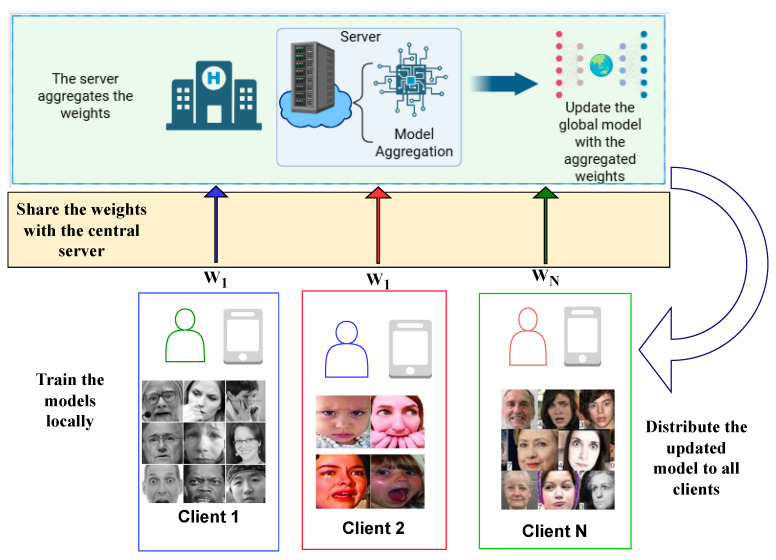
Proposed FL-based system for privacy-preserving FER.

In this paper, we propose a privacy-preserving federated learning-based facial emotion recognition framework designed for assistive technology. Federated learning is incorporated to ensure data privacy and enhance the model’s generalization across diverse users and devices. The framework employs a lightweight deep learning model optimized for edge deployment, enabling real-time performance while maintaining privacy. FL is used to implement collaborative, iterative training across multiple devices. To emulate real-world scenarios where client data distributions are inherently heterogeneous with variations in ethnicity and demographic characteristics. Three popular FER datasets, FER2013, RAF-DB, and ExpW, were used for training and testing in a heterogeneous data environment. These datasets include naturally captured facial samples with diverse demographic and environmental characteristics. Figure 1 shows the high-level overview of the proposed system.

The FL setup involves multiple clients and a central server. Each client trains a local copy of the model using its own data and sends only the model updates to the server. The server then aggregates these updates from all clients to produce an improved global model. This process repeats over several rounds until the model converges.

### 3.3. Datasets

To evaluate the proposed model, each of the three datasets utilized in this research contributes complementary heterogeneity in demographics, environment, and situational context:

- Real-world Affective Faces Database (RAF-DB) [54]: contributes a total of 12,488 images split into 9983 training and 2505 test samples, capturing a wide demographic range with variations in age, ethnicity, gender, head poses, and illumination. The dataset follows its official split of approximately 80:20 (train:test), which we retained to ensure comparability with prior works.
- Facial Emotion Recognition 2013 (FER2013) [55]: provides 16,482 images under challenging in-the-wild conditions such as occlusions and variable lighting, split into 13,269 training and 3213 test samples (80:20).
- Expression in the Wild (ExpW) [56]: adds situational diversity with 79,650 images of both posed and spontaneous expressions, split into 55,739 training and 23,911 test samples (70:30).

Together, these datasets provide complementary demographic, environmental, and situational heterogeneity for training and evaluating robust FER models.

### 3.4. Explainability, Performance, and Trustworthiness-Driven CNN Architecture Optimization

To identify the best lightweight architecture for facial emotion recognition, a number of CNN models with different filter configurations were trained and evaluated within a federated learning framework. Several lightweight CNN architectures were examined with different filter block configurations. For the three-layer models, the configurations included (16, 32, 64), (32, 64, 128), (64, 128, 256), and (128, 128, 256), while the four-layer models used (16, 32, 64, 128), (32, 64, 128, 256), and (64, 128, 256, 512). The models under evaluation have parameter counts ranging from 164 K to 1.72 M, encompassing both ultra-lightweight and lightweight designs. This range allows for direct comparison with edge-optimized architectures like MobileNetV2 (3.5 M parameters, ≈14 MB) [57], SqueezeNet (1.24 M parameters), and ShuffleNet (1.0–2.3 M parameters) [58].

Using Grad-CAM++, we quantitatively evaluated the explanation quality of our lightweight architectures on the RAF-DB, ExpW, and FER2013 datasets. While most evaluations rely on standard performance metrics such as accuracy or on qualitative visual inspection of model interpretations, our approach utilized a quantitative assessment strategy for model explainability. The final configuration was selected based on both its strong performance and the reliability of its explanations.

**Figure 2:**
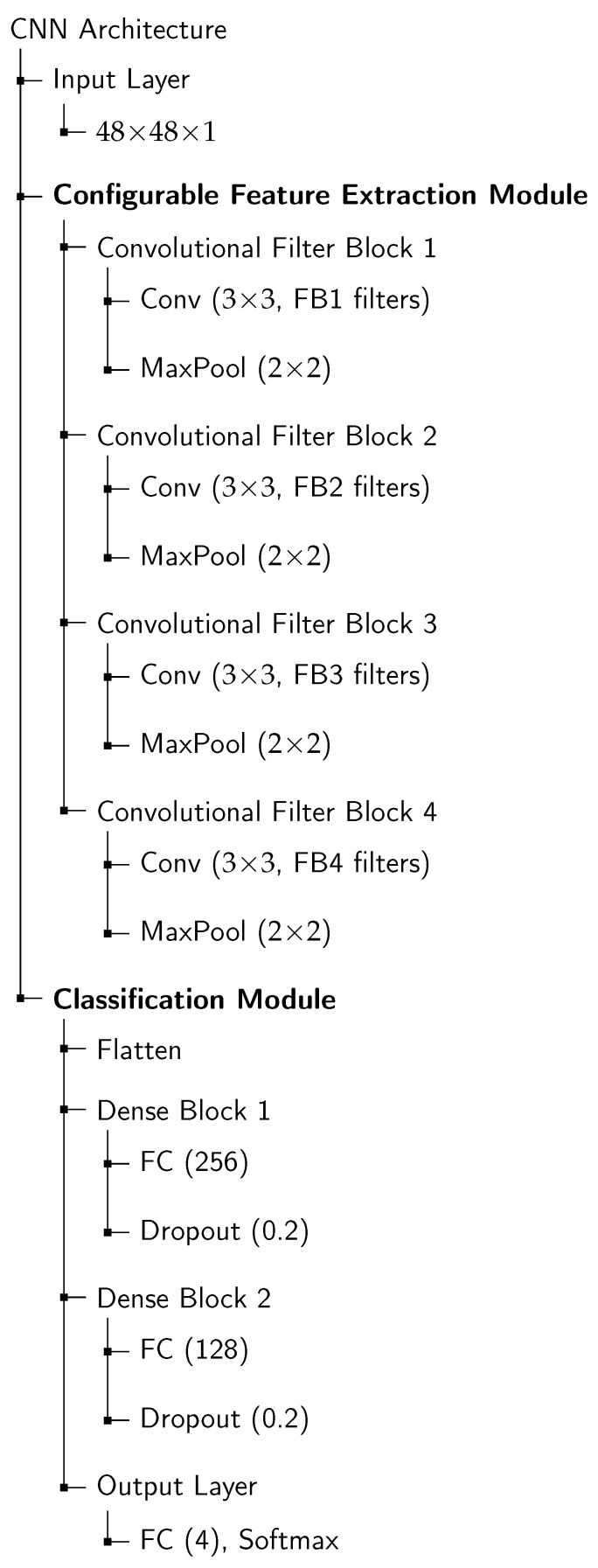
Hierarchical CNN architecture for facial emotion recognition. The Feature Extraction Module is configurable via the filter sizes (FB1–FB4).

The general architecture of the evaluated CNN models is illustrated in Figure 2. The network processes 48×48 grayscale facial images using a variable number of convolutional filter blocks, each consisting of a 3×3 convolutional layer followed by 2×2 max-pooling. The configurable filter depths (FB1–FB4) correspond to the different filter configurations evaluated across the three-layer and four-layer architectures. After feature extraction, the feature maps are flattened and passed through two fully connected layers with 256 and 128 units, followed by a dropout layer (rate = 0.2) for regularization. The final output layer employs a softmax activation function to classify the four target emotions: neutral, happy, sad, and angry.

Training samples from the FER2013, RAF-DB, and ExpW datasets were merged and shuffled to emulate realistic heterogeneous data distributions. The combined dataset was then distributed among three clients in the federated training setup. Each client trained its local model for 15 epochs per round using a batch size of 64 and a learning rate of 0.001. At the end of each round, the clients transmitted their model weights to a central server, where aggregation was performed using the Federated Averaging (FedAvg) [59] algorithm. Model performance across all configurations was evaluated using accuracy, F1-score, recall, and precision. Training continued until the global model converged, when performance stabilized without any further notable improvement. This occurred after approximately 20 rounds.

#### Grad-CAM++ Explainability Evaluation

To assess the explainability and trustworthiness of the CNN architectures, Grad-CAM++ [60] was employed as the primary explanation method, followed by quantitative evaluation using perturbation-based metrics. Grad-CAM++ generates class-specific heatmaps that highlight the regions most influential to the model’s predictions by weighting feature maps with higher-order gradient information. The resulting activation maps were normalized to the range [0, 1] for quantitative analysis.

To compare interpretability across the candidate CNN architectures and to examine the contribution of each convolutional layer to the decision-making process, Grad-CAM++ overlays were generated for each convolutional block: Filter Block 1–Filter Block 3 (FB1–FB3) for the three-layer models and Filter Block 1–Filter Block 4 (FB1–FB4) for the four-layer model. The visualizations were produced using images correctly classified by all models from the FER2013, RAF-DB, and ExpW datasets.

For the final overlay, the Grad-CAM++ heatmap was combined with a channel-attention map to enhance localization consistency and suppress irrelevant activations. This integration, inspired by the Convolutional Block Attention Module (CBAM) [61], allows the network to simultaneously detect the important features (channel attention) and where they occur (spatial attention), thereby producing more informative and interpretable feature representations. All preprocessing steps and parameters were maintained consistently across all models.

### 3.5. Assessing Grad-CAM++ Explanations with Perturbation-Based Metrics

To systematically evaluate the faithfulness of the Grad-CAM++ explanations, we employed a set of perturbation-based quantitative metrics that measure how the model’s prediction changes under controlled modifications of the input image. These metrics provide an objective way to determine whether the highlighted regions in the explanation map truly influence the model’s decision-making. In particular, we employ three widely adopted families of perturbation-based metrics:

- Insertion and Deletion AUC (IAUC/DAUC), which quantify how the model’s confidence evolves as the most salient regions are progressively revealed or removed.
- Average Drop (AD) and Average Drop in Accuracy (ADA), which measure the reduction in confidence and accuracy when the important regions highlighted by the explanation are removed.
- Increase in Confidence (IC), which counts cases where retaining only the salient regions leads to higher confidence than using the full image.

The full mathematical definitions and implementation details for all metrics are provided in Appendix A.

## 4. Results Analysis and Discussion

### 4.1. Optimization and Selection of CNN Architectures for Explainability Analysis

This subsection presents the optimization results of various CNN architectures, comparing them across the RAF-DB, ExpW, and FER2013 datasets. The main goal is to find the top-performing models that exhibit good generalization across these diverse datasets, as these will make solid candidates for further assessment of the quality of the explanation.

The performance of the three-layer and four-layer CNN architectures under various filter configurations is presented in Table 1, Table 2, Table 3 and Table 4, Table 5, Table 6, respectively. These tables report the accuracy, F1-score, recall, and precision for each configuration, with bold indicating the best-performing configuration for each metric.

Among the three-layer CNNs, two configurations showed improved performance. With an accuracy of 82.8% on RAF-DB, the (64, 128, 256) model outperformed the (128, 128, 256) model, which came in second with 80.7%. Both configurations attained similar accuracy values of 71.1% on FER2013, while (128, 128, 256) performed marginally better on ExpW. Furthermore, the (64, 128, 256) model demonstrated consistent improvements across all other metrics, including F1-score, recall, and precision. Considering the goal of identifying models that exhibit both high accuracy and strong generalization across diverse datasets, the (64, 128, 256) configuration was selected for further analysis of its explainability. Among the four-layer CNNs, the (64, 128, 256, 512) configuration achieved the best overall results, recording the highest values across all metrics on both RAF-DB and ExpW. It reached an accuracy of 80.6% on RAF-DB and 69.0% on ExpW, while maintaining comparable performance on FER2013 with 70.4%.

**Figure 3:**
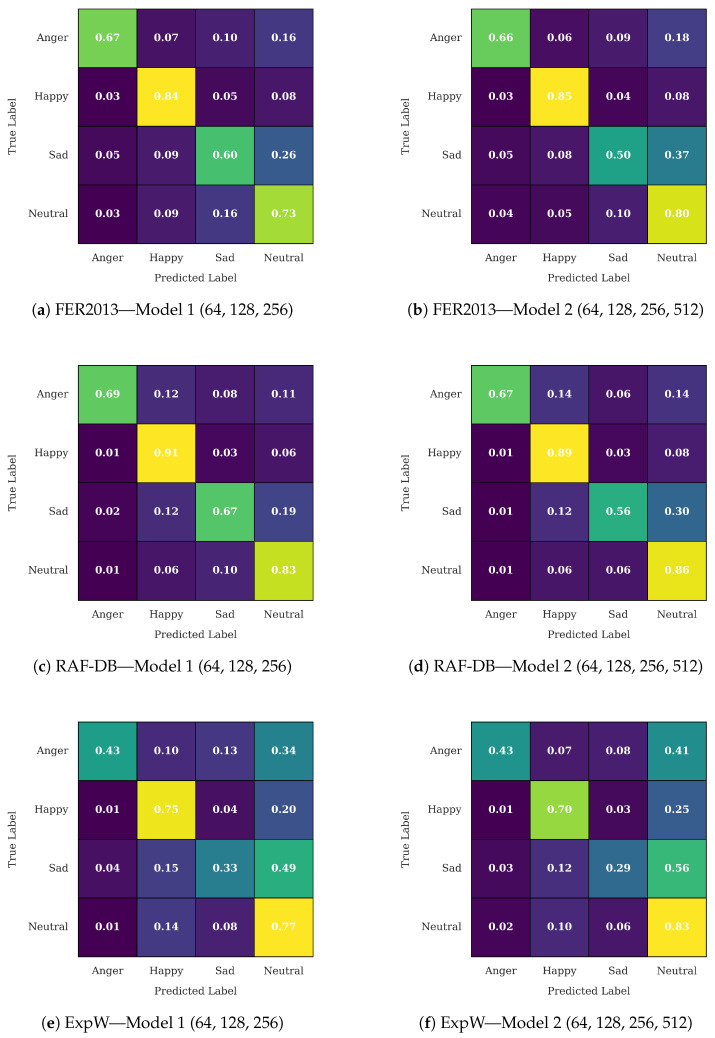
Normalized confusion matrices for Model 1 and Model 2 across FER2013, RAF-DB, and ExpW datasets.

Incorporating the best model from each configuration allows for a more thorough examination of how interpretability is affected by increasing filter depth. Models that generalize well across datasets typically generate more targeted and semantically meaningful activation patterns, which further emphasizes the relationship between interpretability and accuracy. The confusion matrix for the selected model is shown in Figure 3. Overall, both architectures demonstrated comparable performance across datasets. However, the three-layer model showed enhanced discrimination for negative emotions such as sad and anger, while the four-layer model performed slightly better on neutral and happy expressions.

To further examine interpretability and understand the contribution of each convolutional component, Grad-CAM++ overlays were generated for each Filter Block (FB): FB1–FB3 for the three-layer model and FB1–FB4 for the four-layer model, as illustrated in Table 7. The filter block-wise heatmaps show a consistent interpretability progression within the three-layer model. Low-level facial edges were mainly captured by FB1, whereas mid-level semantic areas like the corners of the mouth, eyes, and eyebrows were prioritized by FB2. Effective spatial focus and discriminative learning were demonstrated by FB3, which generated highly class relevant activation regions.

In the four-layer model, a similar progression was observed across FB1–FB3; however, the additional FB4 produced diffuse activations that extended beyond salient facial regions. The overlay visualizations showed spatially dispersed responses and no clear gradient patterns in FB4’s feature maps, indicating weakened gradient flow and reduced semantic focus. These effects likely result from gradient degradation and overfitting caused by the extra depth and capacity, leading to redundant rather than informative features. This observation aligns with the findings of [32,62], which report that increasing model complexity often reduces the spatial accuracy and causal relevance of deeper layer explanations.

In order to assess interpretability and explainability of the best chosen models, Grad-CAM++ visualizations obtained from the third convolutional layer (FB3) were used to evaluate interpretability and explainability, as deeper layers generally exhibit reduced spatial precision and less meaningful activations [62]. The Grad-CAM++ overlays of the corresponding layers are shown in Table 8. The results demonstrate that both the (64, 128, 256) and (64, 128, 256, 512) models have the ability to highlight relevant facial features for the given emotions as demonstrated by the activation maps. Both models are able to give meaningful visual explanations for their predictions by consistently highlighting the key features for each emotion, such as furrowed eyebrows and tight lips for anger, drooping mouth and lowered eyelids for sadness, smiling mouth and eyes for happiness, and more diffuse activation maps for neutral expressions.

The (64, 128, 256) model generally produced more meaningful heatmaps compared to the (64, 128, 256, 512) model, suggesting better overall explainability, especially for anger and sad samples. However, for some happy and neutral samples, the (64, 128, 256, 512) model’s activation maps appear to be more concentrated on key facial regions. This aligns with the confusion matrix analysis that concluded that the (64, 128, 256) model performs better on anger and sad emotions, while the (64, 128, 256, 512) model performs slightly better on happy and neutral emotions.

The results indicate that the (64, 128, 256) model demonstrates superior overall explainability. However, a more extensive analysis on a larger dataset is required to thoroughly assess the generalizability and consistency of the model visualizations. To quantitatively evaluate the explainability of the models, a comprehensive assessment was conducted using perturbation-based metrics, including IAUC, DAUC, AD, ADA, and IC, computed on the Grad-CAM++ saliency maps. Higher IAUC, AD, ADA, and IC values, along with lower DAUC scores, reflect more faithful and relevant explanations. This quantitative approach complements the qualitative visual inspection and provides a systematic comparison of the models’ explainability across multiple datasets. It thereby supports more definitive conclusions regarding their consistency, generalizability, and trustworthiness for facial emotion recognition. The metrics were evaluated on 17,739 samples, combining the FER2013, RAF-DB, and ExpW test sets, where both models were assessed. Table 9 summarizes the performance comparison of the (64, 128, 256) and (64, 128, 256, 512) models across the different explainability metrics.

For the anger and sad emotions, the (64, 128, 256) model consistently outperforms the (64, 128, 256, 512) model across nearly all explainability metrics. It achieves higher IAUC, AD, IC, and ADA values, along with lower active pixel ratios and post-masking accuracy, reflecting stronger causal relevance and more focused saliency distributions. Although the 4-layer model exhibits slightly lower DAUC values, the difference is marginal. The slightly higher DAUC observed in the 3-layer model indicates a slower confidence drop when key regions are removed, suggesting reliance on broader yet still meaningful feature activations. This behavior highlights the model’s ability to capture semantically relevant but spatially distributed cues, enhancing both robustness and interpretability. Overall, the (64, 128, 256) model demonstrates superior spatial coherence and explanatory consistency for both anger and sad emotions.

For the Happy emotion, the (64, 128, 256) model achieves higher IAUC and IC scores, along with lower DAUC and Active Pixel ratio compared to the (64, 128, 256, 512) model. These results indicate that the shallower model provides more concentrated and discriminative activation regions, focusing more effectively on the key facial cues that characterize happiness. Although the deeper model shows slightly higher AD and ADA values, suggesting broader activations, leading to a higher performance drop after masking, as they include widespread but less informative regions. This is supported by the higher active pixel ratio of 0.578, confirming that the deeper model activates larger portions of the face. In other words, the deeper model captures more extensive areas with reduced localization, making its explanations appear more diffuse. Overall, the 3-layer model produces clearer and more interpretable attention patterns, while the 4-layer model tends to capture stronger but less focused responses.

The (64, 128, 256, 512) model outperforms the (64, 128, 256) model in terms of IAUC and IC for the neutral emotion, suggesting more reliable activation when detecting neutral features. However, it shows higher DAUC and lower ADA, suggesting that the deeper model captures stronger responses; its activations are less spatially precise when features are removed. The relatively high active pixels of 0.510 vs. 0.451 active pixels of both models reflect the difficulty of identifying neutral expressions, which lack distinctive cues and frequently cause the model to activate broader and less discriminative areas in order to rule out emotional features rather than focus on specific facial indicators.

Overall, the comparative analysis indicates that the (64, 128, 256) model offers a better balance between performance and interpretability across emotions. Table 10 summarizes the comparative metrics and supports this observation, showing that the model consistently achieves higher IAUC, IC, and ADA scores and lower DAUC and Active Pixel ratios for most emotions. These findings confirm its ability to generate more compact and discriminative attention maps. Even for the neutral emotion, where the deeper model shows stronger global activations, the shallower model maintains competitive performance with clearer spatial focus. The slightly worse values in some deletion-based metrics, such as DAUC and AD for Happy and Sad, are likely due to the model’s lower activation ratio, as it focuses on a smaller number of key regions compared to deeper models that remove more pixels, which can cause a sharper drop in performance.

**Figure 4:**
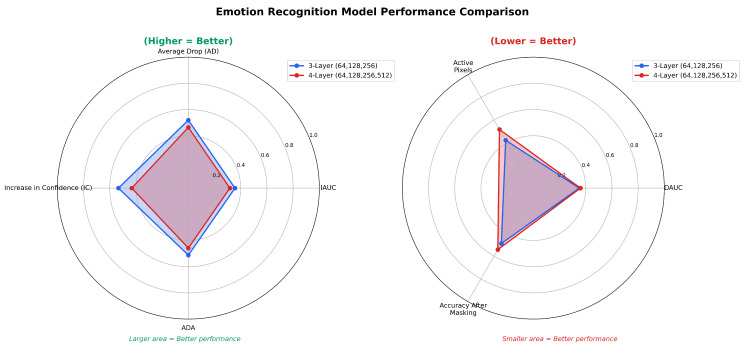
Radar-plot comparison of 3-layer (64, 128, 256) and 4-layer (64, 128, 256, 512) CNN emotion recognition models. (**Left**): higher-is-better metrics (IAUC, AD, IC, ADA). (**Right**): lower-is-better metrics (DAUC, Active Pixels, Accuracy after masking).

Overall, the comparative analysis indicates that the (64, 128, 256) model provides a better balance between performance and interpretability across emotions. Table 10 summarizes the comparative metrics and supports this observation, showing that the (64, 128, 256) model achieves a better balance between performance and interpretability across emotions. It consistently achieves higher IAUC, IC, and ADA scores and lower DAUC and active pixel ratios for most emotions, confirming that it generates more compact and discriminative attention maps. Even for the neutral emotion, where the deeper model shows stronger global activations, the shallow model maintains competitive performance with clearer spatial focus. The slightly worse values in some deletion-based metrics, such as DAUC and AD for happy and sad, are likely due to the model’s lower activation ratio as it focuses on a smaller number of key regions compared to the other models that remove more pixels that can cause a sharper drop in performance. To ensure the reliability of these findings, the obtained Grad-CAM++ explainability metrics for all emotion classes were compared against empirically observed ranges reported in prior works. Average AD values typically range from 0.195 to 0.598 and IC from 0.067 to 0.191 [60], while IAUC and DAUC span approximately from 0.16 to 0.49 and from 0.016 to 0.296, respectively [63]. These comparisons confirm the stability and interpretability validity of the evaluated models. Figure 4 illustrates the comparative distribution of Grad-CAM++ explainability metrics. The (64, 128, 256) model exhibits superior explainability, showing higher AD, IAUC, ADA, and IC values, reflected by larger radar areas, while achieving lower DAUC, masked accuracy, and active pixel ratio, indicated by smaller radar areas for relevant metrics.

### 4.2. Reproducibility and Statistical Evaluation

To ensure the reliability and reproducibility of our federated learning experiments, the reported results for the FedXAI were computed over ten random seeds {1, 3, 7, 17, 21, 33, 42, 55, 77, 100} and evaluated on the three datasets. This multi-seed evaluation captures stochastic variability originating from model initialization, batch sampling, and client update ordering. For each metric, we report the mean and standard deviation (Std) across runs. The proposed framework’s robustness and stability were measured using a 95% confidence interval (95% CI) over repeated trials. Table 11 shows consistently narrow confidence intervals and low standard deviations across the datasets confirming the stable and statistically reliable performance of the proposed FedXAI framework.

### 4.3. Discussion on Client Scaling and Data Characteristics

To ensure a fair comparison between model configurations, we adopt a federated setup where all clients receive an equal number of samples and the same emotion classes, using a shuffled combination of the three FER datasets to introduce a level of heterogeneity. This controlled design ensures that any variation in performance can be attributed directly to architectural and configuration differences rather than fluctuations in client or data distribution. The integration of three natural FER datasets introduces inherent variability in capture conditions and label characteristics, providing a form of non-IID behavior across clients. To further examine how the framework behaves under increasingly distributed conditions, we conducted additional experiments by varying the number of participating clients. This analysis allows us to assess how performance scales as data become more distributed across a larger number of clients. Table 12 shows that the model maintains consistent accuracy across 3, 6, 9, and 15 clients, with only small fluctuations across configurations, which is expected given the increase in data fragmentation and heterogeneity. Despite this, the model exhibits stable performance under moderate scaling.

However, real-world federated environments typically involve a larger number of clients, higher degrees of data heterogeneity, and stronger non-IID conditions, which remain challenging for practical deployment. In future work, we will expand the framework to larger-scale settings and consider aggregation strategies tailored to more challenging non-IID and heterogeneous client scenarios.

## 5. Comparison with Cross-Dataset Evaluation Methods

To demonstrate the generalization capability of the proposed framework, we benchmark it against representative cross-dataset FER approaches on RAF-DB, ExpW, and FER2013. Unlike methods trained on a single dataset, these approaches are designed to operate across heterogeneous data sources. For fair evaluation, we also train our network in a centralized setup by combining and shuffling the three datasets and follow the same test protocol as in the federated configuration. Table 13 reports the performance of state-of-the-art cross-dataset FER models spanning both large-scale and lightweight architectures, as established in [64]. While large backbone networks such as ResNet-50 variants achieve competitive results, several methods, including Stepwise Adaptive Feature Norm (SAFN) [65], Conditional Adversarial Domain Adaptation (CADA) [66], and Enhanced Channel Attention Network (ECAN) [67], exhibited limited generalization, particularly on FER2013. Though attention-based models, such as Attention-Guided Residual Aggregation (AGRA) [68], Cross-Global Local Representation Learning (CGLRL) [69], and Label Dynamic Weight Matching (LDWM) [64], improve cross-domain learning, however, they show noticeable performance drops across the different test sets.

In contrast, our lightweight convolutional architecture delivers the highest mean accuracy (75.5%) among all lightweight models and even surpasses all ResNet-50 based cross-domain baselines in average accuracy. Moreover, it achieves consistently strong results across datasets, including the challenging FER2013 benchmark, which many methods struggled with.

Importantly, the federated version of our model sustained strong performance under data heterogeneity, achieving 82.8% (RAF-DB), 68.8% (ExpW), and 71.3% (FER2013), with an overall average of 74.3%. The marginal 1.2% gap compared to centralized training highlights the robustness and efficiency of our federated optimization pipeline, confirming the feasibility of privacy-preserving facial emotion recognition on distributed edge devices.

Overall, these results establish our method as a practical and scalable solution for real-world FER, effectively balancing accuracy, computational efficiency, and privacy. The superior mean accuracy across datasets, combined with compact model size and stable cross-domain performance, positions the proposed framework as a strong candidate for potential deployment in assistive and resource-constrained environments.

## 6. Edge Deployment and Runtime Evaluation

The real-world potential deployment feasibility was validated using a Raspberry Pi 4 Model B equipped with a 1.5 GHz quad-core ARM Cortex-A72 processor and 8 GB RAM. After conversion to TensorFlow Lite for optimized edge inference, the model was evaluated on the FER2013 test set and demonstrated excellent runtime performance, as shown in Table 14. The system achieved an average inference latency of 13.92 ± 1.46 ms, corresponding to 71.84 FPS throughput, exceeding the 15–30 FPS requirement for real-time video analysis [70]. Resource usage remained low, consuming only 25.30 ± 8.51% of a single CPU core while maintaining a peak memory footprint of 156.10 MB. Importantly, the optimized model retained its classification accuracy at 71.1%. With a compact storage requirement of 5.5 MB and a runtime memory overhead of 1.00 MB, demonstrating that the model is highly suitable for resource-constrained edge environments.

A comparative analysis further underscores the advantages of the proposed lightweight CNN. For instance, MobileNetV2 requires 513.6 ms per inference (approximately 2 FPS) on the same hardware [71] with 25.6% CPU utilization, whereas our model achieves significantly lower inference latency with a comparable CPU usage of 25.3%. The proposed system also outperforms Light-FER’s reported throughput of 4.85 FPS [72] and demonstrates improved efficiency over existing Raspberry Pi 4 benchmarks, with MobileNetV1 achieving 82.7 ms inference time and MobileNetV2 reaching 112.6 ms using an accelerator [73].

In comparison with widely used mobile architectures reported in recent FER studies, the efficiency of the proposed lightweight CNN is further supported through its faster inference and higher throughput on edge devices. For example, in [74], MobileNetV1 (4.3 M parameters, 600 M FLOPs) achieved 22.6 ms, whereas MobileNetV2 (3.5 M parameters, 312.86 M FLOPs) reported 25.9 ms on an Intel Xeon Silver 4214R CPU at 2.40 GHz with dual processors and 128 GB RAM. In another study evaluating lightweight CNN models [75], MobileNetV2 with 3.51 M parameters and 0.6016 GFLOPs achieved an inference time of 58.4 ± 3.5 ms, while ShuffleNetV2 with 3.99 M parameters and 0.9743 GFLOPs required 63.3 ± 6.2 ms. Their recognition performance reached 58% and 65% accuracy on FER2013 and 73% and 80% on RAF-DB, respectively. A third study on an improved MobileNetV2 [76] reported 67.9% accuracy on FER2013 with a mobile inference latency of 300 ms on Huawei P60 hardware.

In comparison, the proposed model is significantly lighter and faster, requiring only 1.45 M parameters and 107 MFLOPs, yet achieving 71.1% accuracy on FER2013 and 82.8% on RAF-DB. Moreover, it delivers real-time inference at 13.92 ms on a Raspberry Pi 4, a substantially more resource-constrained edge device than the high-end desktop systems or modern smartphones used in the prior studies. Structurally, the proposed network uses three convolutional layers with a principled (64, 128, 256) channel progression that utilizes hierarchical feature learning [77], in which each layer increases representational capacity while maintaining computational efficiency suitable for edge devices. This design avoids the architectural complexity of MobileNetV1, MobileNetV2, and ShuffleNetV2, which rely on deeper architectures with numerous depthwise-separable convolutions, inverted residual blocks, and channel-shuffle units that introduce additional overhead and reduce interpretability. The three-layer structure provides efficient feature extraction while preserving spatial locality, which is crucial for facial emotion recognition [78], making it particularly suitable for edge deployment where computational resources and power budgets are constrained [79].

## 7. Ethical and Practical Considerations for Deployment

Several practical and ethical issues must be considered for potential deployment of FER systems in mental health contexts. Publicly available datasets are not designed for clinical assessment and do not represent clinical contexts, as a result limiting their suitability to such applications [5]. Furthermore, expert evaluation is essential for trustworthy deployment of AI systems in clinical contexts to confirm the alignment of the model-highlighted regions with meaningful and relevant cues [5]. According to [80], both individual AI model decisions and overall system behavior should be open to review by auditors. Future work will incorporate qualitative, expert-driven assessment of explanations in collaboration with mental health professionals to evaluate whether the highlighted regions correspond to clinically meaningful cues and to assess the practical interpretability of the system in real clinical contexts.

Bias is another major ethical challenge, occurring when FER systems make discriminatory decisions that disadvantage certain groups due to dataset imbalance, cross-cultural differences in emotional expression, and variations associated with mental health conditions [5]. Bias can also arise at multiple stages of the AI pipeline, including annotation practices, modeling choices, and deployment processes [80].

As a result, bias mitigation should involve multi-level strategies at the data, algorithmic, and evaluation levels. At the data level, recommended practices include demographic auditing, balanced sampling, and ensuring adequate representation of diverse and clinically relevant groups [81,82], as emphasized in recent fairness and bias mitigation surveys [83,84]. At the algorithmic level, explainability methods help reveal biased decision patterns [82]. In addition, fairness-aware modeling approaches include sampling adjustments, transparency mechanisms, and mitigation techniques such as reweighting, adversarial debiasing, equalized-odds post-processing, and fair representation learning, which aim to reduce unfair performance differences across demographic groups [80]. At the evaluation level, ongoing fairness monitoring across demographic subgroups is essential for detecting performance disparities [5]. Independent auditing frameworks are also recommended to support accountability and oversight [80].

Although federated learning prevents raw data sharing, privacy risks such as inference attacks or leakage through model updates remain an ongoing concern. Prior work on emotion-recognition technologies suggests that these systems are also susceptible to unintended inferences or misuse of derived information [5,82]. As a result, additional safeguards, including secure aggregation, differential privacy, and transparent auditing, are important components of responsible deployment. Given the sensitivity of emotional and mental health related information, such protections are essential for safe deployment [85].

Informed consent is another important consideration for continuous emotion monitoring in mental health contexts. FER systems may involve ongoing or repeated data processing, which raises additional considerations for meaningful informed consent [82]. To support meaningful consent, systems should provide clear and accessible explanations of data use, model limitations, and the scope of analysis, along with user controls that allow individuals to pause or disable monitoring. These steps are aligned with recent recommendations for ethically deploying affective computing technologies [5]. Informed consent is another important consideration for continuous emotion monitoring in mental health contexts. FER systems involve ongoing and repeated data processing, which raises additional requirements for meaningful informed consent [82]. To support informed consent, systems should provide clear and accessible explanations of data use, model limitations, and the scope of analysis, along with user controls that allow individuals to pause or disable monitoring, in line with recent recommendations for ethically deploying affective computing technologies [5].

Accountability and transparency are also essential for responsible deployment. At the technical level, documentation such as model cards, which provide standardized information on a model’s purpose, data sources, evaluation metrics, and known limitations, can help clinicians and users understand system behavior [82]. Recent best-practice guidelines emphasize structured documentation, including dataset provenance and model-level reporting, to support transparency and enable external auditing [80]. At the clinical level, human oversight remains crucial, with clinicians reviewing AI-generated outputs and retaining the authority to override system recommendations. Explainability methods used in this work support accountability by enabling domain experts to inspect the basis of model decisions. Together, these practices help ensure that FER-based mental health tools are deployed safely and responsibly [5].

## 8. Conclusions

This work presented a federated and explainability-driven facial emotion recognition framework that enables trustworthy, privacy-preserving, and edge-deployable emotion analysis with potential applications in mental health monitoring. Unlike traditional centralized FER systems, the proposed approach preserves data confidentiality through federated learning while enhancing transparency through quantitative evaluation of explanation quality using Grad-CAM++ perturbation-based metrics, including IAUC, DAUC, AD, IC, ADA, and Active Pixel Ratio.

A lightweight CNN architecture was designed to achieve efficient training and inference on resource-constrained devices while maintaining high accuracy. Extensive experiments on RAF-DB, ExpW, and FER2013 datasets demonstrated that the proposed model achieved strong cross-dataset generalization among lightweight architectures, with 75.5% and 74.3% average accuracies under centralized and federated configurations, respectively, confirming the framework’s generalization and suitability for potential decentralized deployment.

By integrating interpretability into the model selection process, this study establishes a quantitative and transparent pathway for trustworthy FER systems. Future work will extend this framework toward personalized federated learning and temporal emotion modeling, enabling continuous and adaptive affective monitoring in real-world assistive and mental health contexts. Additionally, integrating multimodal data sources, such as audio cues, physiological signals, and contextual information, will enable more comprehensive and contextually aware emotion understanding. Future work will also include qualitative validation of the Grad-CAM++ explanations, in collaboration with mental health professionals, to establish clinical interpretability.

## Figures and Tables

**Table 1:** Performance comparison of CNN (3-layer) models on FER2013 dataset.

Metric	(16, 32, 64)	(32, 64, 128)	(64, 128, 256)	(128, 128, 256)
Accuracy	0.675	0.704	0.711	0.711
F1 Score	0.673	0.702	0.712	0.709
Recall	0.675	0.704	0.711	0.711
Precision	0.688	0.712	0.723	0.719

**Table 2:** Performance comparison of CNN (3-layer) models on RAF-DB dataset.

Metric	(16, 32, 64)	(32, 64, 128)	(64, 128, 256)	(128, 128, 256)
Accuracy	0.778	0.813	0.828	0.807
F1 Score	0.775	0.812	0.827	0.804
Recall	0.778	0.813	0.828	0.807
Precision	0.774	0.812	0.828	0.807

**Table 3:** Performance comparison of CNN (3-layer) models on ExpW dataset.

Metric	(16, 32, 64)	(32, 64, 128)	(64, 128, 256)	(128, 128, 256)
Accuracy	0.665	0.690	0.688	0.691
F1 Score	0.658	0.681	0.681	0.681
Recall	0.665	0.690	0.688	0.691
Precision	0.665	0.680	0.679	0.682

**Table 4:** Performance comparison of CNN (4-layer) models on FER2013 dataset.

Metric	(16, 32, 64, 128)	(32, 64, 128, 256)	(64, 128, 256, 512)	(128, 128, 256, 512)
Accuracy	0.679	0.696	0.704	0.716
F1 Score	0.678	0.695	0.702	0.715
Recall	0.679	0.696	0.704	0.716
Precision	0.685	0.705	0.723	0.719

**Table 5:** Performance comparison of CNN (4-layer) models on RAF-DB dataset.

Metric	(16, 32, 64, 128)	(32, 64, 128, 256)	(64, 128, 256, 512)	(128, 128, 256, 512)
Accuracy	0.760	0.785	0.806	0.797
F1 Score	0.757	0.784	0.802	0.795
Recall	0.760	0.785	0.806	0.797
Precision	0.756	0.784	0.811	0.794

**Table 6:** Performance comparison of CNN (4-layer) models on ExpW dataset.

Metric	(16, 32, 64, 128)	(32, 64, 128, 256)	(64, 128, 256, 512)	(128, 128, 256, 512)
Accuracy	0.649	0.676	0.690	0.682
F1 Score	0.647	0.669	0.679	0.676
Recall	0.649	0.676	0.690	0.682
Precision	0.645	0.666	0.687	0.675

**Table 7:** Layer-wise Grad-CAM++ visualizations for the selected models, showing activation progression across feature blocks from shallow (FB1) to deep (FB4) layers.

Model	Emotion	Sample	Grad-CAM++ Overlays			
			**FB1**	**FB2**	**FB3**	**FB4**
(64, 128, 256)	Anger	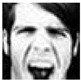	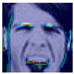	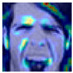	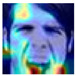	—
(64, 128, 256, 512)	Anger	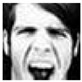	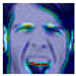	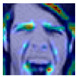	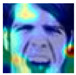	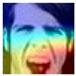
(64, 128, 256)	Anger	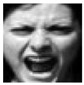	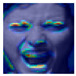	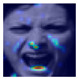	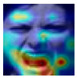	—
(64, 128, 256, 512)	Anger	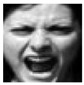	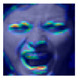	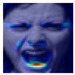	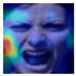	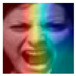
(64, 128, 256)	Sad	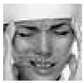	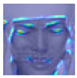	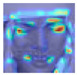	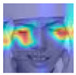	—
(64, 128, 256, 512)	Sad	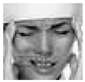	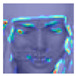	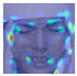	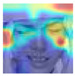	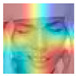
(64, 128, 256)	Sad	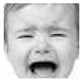	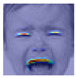	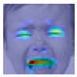	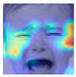	—
(64, 128, 256, 512)	Sad	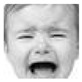	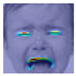	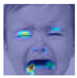	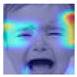	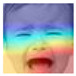
(64, 128, 256)	Happy	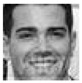	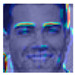	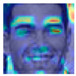	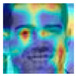	—
(64, 128, 256, 512)	Happy	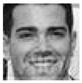	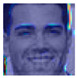	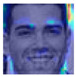	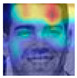	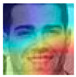
(64, 128, 256)	Happy	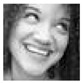	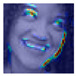	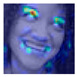	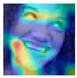	—
(64, 128, 256, 512)	Happy	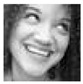	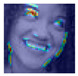	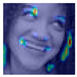	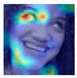	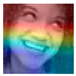

**Table 8:** Qualitative examples of Grad-CAM++ overlays extracted from the third convolutional block (FB3), demonstrating spatial focus patterns and interpretability characteristics for the evaluated models.

Emotion	Sample	Grad-CAM++ Overlays (FB3)	
		**(64, 128, 256)**	**(64, 128, 256, 512)**
Anger	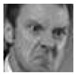	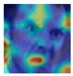	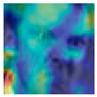
Anger	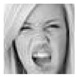	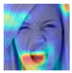	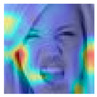
Sad	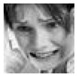	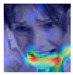	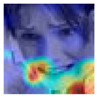
Sad	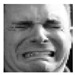	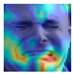	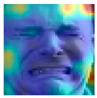
Happy	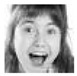	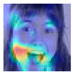	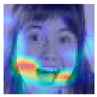
Happy	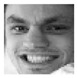	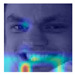	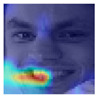
Neutral	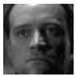	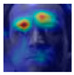	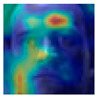
Neutral	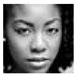	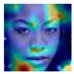	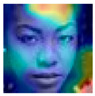

**Table 9:** Comparison of Grad-CAM++ metrics for all emotions across the models. Where (↑) and (↓) are indicators of higher-is-better and lower-is-better metrics, respectively.

Metric	Anger		Happy		Sad		Neutral	
	**(64, 128, 256)**	**(64, 128, 256, 512)**	**(64, 128, 256)**	**(64, 128, 256, 512)**	**(64, 128, 256)**	**(64, 128, 256, 512)**	**(64, 128, 256)**	**(64, 128, 256, 512)**
Original Confidence (↑)	0.982	0.972	0.992	0.965	0.970	0.912	0.962	0.918
IAUC (↑)	0.226	0.191	0.268	0.145	0.230	0.154	0.699	0.777
DAUC (↓)	0.258	0.230	0.211	0.247	0.263	0.197	0.672	0.765
AD (↑)	0.708	0.557	0.369	0.456	0.690	0.667	0.308	0.177
IC (↑)	0.436	0.222	0.519	0.355	0.414	0.234	0.768	0.920
ADA (↑)	0.713	0.563	0.331	0.422	0.702	0.674	0.303	0.172
Active Pixels (↓)	0.401	0.481	0.373	0.547	0.464	0.529	0.451	0.510
Accuracy After Masking (↓)	0.287	0.437	0.669	0.578	0.298	0.326	0.697	0.828

**Table 10:** Average performance comparison of (64, 128, 256) and (64, 128, 256, 512) architectures across emotion categories of explainability metrics. Where (↑) and (↓) are indicators of higher-is-better and lower-is-better metrics, respectively.

Model	Original (↑)	IAUC (↑)	DAUC (↓)	AD (↑)	IC (↑)	ADA (↑)	Active (↓)	Accuracy (↓)
	Confidence						Pixels	After Masking
(64, 128, 256)	0.976	0.356	0.351	0.519	0.534	0.512	0.422	0.488
(64, 128, 256, 512)	0.942	0.317	0.360	0.464	0.432	0.458	0.517	0.542

**Table 11:** Statistical evaluation across 10 seeds: mean, standard deviation, and 95% confidence intervals for Accuracy, F1, Recall, and Precision (all reported in %).

Dataset	Metric	Mean	Std	95% CI (Lower)	95% CI (Upper)
FER2013	Accuracy	71.59	0.37	71.33	71.86
	F1	71.52	0.36	71.27	71.78
	Recall	71.59	0.37	71.33	71.86
	Precision	72.30	0.34	72.05	72.55
ExpW	Accuracy	68.82	0.29	68.61	69.03
	F1	68.09	0.25	67.91	68.27
	Recall	68.82	0.29	68.61	69.03
	Precision	68.00	0.33	67.76	68.24
RAF-DB	Accuracy	81.24	0.68	80.75	81.72
	F1	81.06	0.69	80.56	81.55
	Recall	81.24	0.68	80.75	81.72
	Precision	81.16	0.76	80.61	81.70

**Table 12:** Accuracy Across Client Configurations Using the (64, 128, 256) CNN.

Dataset	Clients	Accuracy (%)
FER2013	3	71.99
	6	70.12
	9	70.68
	15	70.25
	Mean	70.76
	Std	0.85
RAF-DB	3	80.80
	6	81.44
	9	80.92
	15	80.24
	Mean	80.85
	Std	0.49
ExpW	3	68.95
	6	68.30
	9	67.71
	15	66.86
	Mean	67.95
	Std	0.89

**Table 13:** Cross-dataset FER comparison on RAF-DB, ExpW, and FER2013.

Method	Backbone	RAF-DB	ExpW	FER2013	Avg.
Large-Scale Architectures					
SAFN [65]	ResNet-50	62.8	64.9	55.6	61.1
CADA [66]	ResNet-50	66.0	63.2	57.6	62.3
ECAN [67]	ResNet-50	53.4	47.4	56.5	52.4
AGRA [68]	ResNet-50	67.6	68.5	59.0	65.0
CGLRL [69]	ResNet-50	71.8	70.0	59.3	67.0
LDWM [64]	ResNet-50	75.9	73.3	61.1	70.1
Lightweight Architectures					
SAFN [65]	MobileNet-V2	38.7	61.4	49.9	50.0
CADA [66]	MobileNet-V2	53.2	59.4	49.3	54.0
ECAN [67]	MobileNet-V2	42.3	45.1	45.8	44.4
AGRA [68]	MobileNet-V2	52.3	64.0	45.8	54.0
CGLRL [69]	MobileNet-V2	62.0	64.9	52.5	59.8
LDWM [64]	MobileNet-V2	64.8	64.9	53.0	60.9
Proposed (Centralized)	Custom	83.6	70.6	72.3	75.5
Proposed (Federated)	Custom	82.8	68.8	71.3	74.3

**Table 14:** Runtime Performance Metrics on Raspberry Pi 4 Model B.

Metric	Value
Model Size	5.5 MB
Average Latency	13.92 ± 1.46 ms
Minimum Latency	12.33 ms
Maximum Latency	18.35 ms
Throughput	71.84 FPS
CPU Usage	25.30 ± 8.51%
Resident Set Size (RSS)	156.10 MB
Steady-State Memory	151.12 MB
Model Size (TFLite)	5.54 MB
MFLOPs per Inference	∼107.4

## Data Availability

The data presented in this study are available on request from the corresponding author.

## Appendix A. Perturbation-Based Metrics

### Appendix A.1. Insertion and Deletion Area Under Curve

In order to assess the faithfulness of saliency maps, we use the two perturbation-based metrics introduced by [86]: Insertion Area Under Curve (IAUC) and Deletion Area Under Curve (DAUC) metrics. This allows us to determine the reliability of the Grad-CAM++ explanations. The metrics measure the changes in the model’s confidence as image regions are gradually added or removed, starting with the most significant features.

Let the input grayscale image be $I\in R^{H\times W}$ and its Grad-CAM++ heatmap be $S\in R^{H\times W}$, normalized to the range [0, 1]. The heatmap *S* is first flattened and sorted in descending order of importance to obtain an ordered index list σ.

At each step *k*, corresponding to revealing or masking a proportion $p_{k}=\frac{k}{N}$ of the total *N* pixels, a new image $I_{k}$ is constructed as follows:

(A1) $$I_{k}^{\left(\mathrm{ins}\right)}\left(x,y\right)=\left\{\begin{array}{cc} I(x,y), & \mathrm{if}\left(x,y\right)\in \sigma_{1:k},\\ 0, & \mathrm{otherwise}, \end{array}\right.$$

(A2) $$I_{k}^{\left(\mathrm{del}\right)}\left(x,y\right)=\left\{\begin{array}{cc} 0, & \mathrm{if}\;\left(x,y\right)\in \sigma_{1:k},\\ I(x,y), & \mathrm{otherwise}, \end{array}\right.$$

where $I_{k}^{\left(\mathrm{ins}\right)}$ denotes the progressively revealed image in insertion and $I_{k}^{\left(\mathrm{del}\right)}$ the progressively occluded image in deletion. At each step, the model’s confidence for the target class *c* is recorded as

(A3) $$f_{k}^{\left(\mathrm{ins}\right)}=m_{c}\left(I_{k}^{\left(\mathrm{ins}\right)}\right),\;f_{k}^{\left(\mathrm{del}\right)}=m_{c}\left(I_{k}^{\left(\mathrm{del}\right)}\right),$$

where $f_{k}^{\left(\mathrm{ins}\right)}$ and $f_{k}^{\left(\mathrm{del}\right)}$ denote the model’s predicted confidence scores for class *c* at step *k* during the insertion and deletion processes, respectively. Here, $m_{c}\left(\cdot \right)$ represents the model’s output probability for the target class *c* given the perturbed image input. IAUC and DAUC are then computed as the area under the corresponding confidence curves. In practice, these integrals are estimated discretely using the trapezoidal rule:

(A4) $$\mathrm{AUC}\approx \sum_{k=1}^{N-1}\frac{\left(f_{k+1}+f_{k}\right)}{2N}.$$

A faithful explanation map should cause a sharp increase in confidence during insertion, yielding a higher IAUC and a sharp decrease in confidence during deletion, yielding a lower DAUC.

### Appendix A.2. Average Drop

A good model captures the important features of an image. When we classify an image, the probability calculated for the correct class is expected to drop if we remove the important regions identified by the explanation map, due to the loss of discriminative features. We use this fact to check the performance of each model by comparing the confidence when the entire image is passed to the classifier versus when the important regions are removed. A better explanation map will identify the most relevant parts, such that removing them leads to a larger drop in confidence. We compute this metric as the average percentage drop in the model’s confidence when the important regions are removed [60,87]. The Average Drop (AD) is expressed as

(A5) $$\mathrm{Average}\;\mathrm{Drop}\;\left(\mathrm{AD}\right)=\frac{1}{N}\sum_{i=1}^{N}\frac{y_{i}^{c}-R_{i}^{c}}{y_{i}^{c}}$$

where

- $y_{i}^{c}$ represents the model’s confidence for class *c* of the *i*th sample when the entire image is used as input.
- $R_{i}^{c}$ is the model’s confidence for class *c* of the *i*th sample when the important regions are removed.
- *N* is the total number of images.

Higher AD values indicate that the explanation successfully identifies decision-critical regions. In addition, we evaluate the Average Drop in Accuracy (ADA), which measures how often the model’s prediction changes when important regions are removed. ADA is defined as

(A6) $$ADA=\frac{1}{N}\sum_{i=1}^{N}\left(1_{\left(P_{i}^{c}=T_{i}^{c}\right)}-1_{\left(R_{i}^{c}=T_{i}^{c}\right)}\right)$$

where:

- 1(·) is the indicator function that equals 1 if the condition is true and 0 otherwise.
- $P_{i}^{c}$ and $R_{i}^{c}$ denote the predicted classes for the original and masked images, respectively.
- $T_{i}^{c}$ represents the true class label for the *i*th sample.

The ADA metric captures the proportion of correctly classified samples that become misclassified after removing the important regions. A higher ADA value implies that the explanation map effectively identifies regions critical for correct classification.

### Appendix A.3. Increase in Confidence

Contrary to previous expectations, there may be scenarios where removing the context helps boost prediction confidence, especially when the context contains noise or unnecessary distractions. Keeping such cases in mind, we calculate the number of times that passing only the explanation map to the classifier increases its confidence compared to passing the entire image.

Formally, the Increase in Confidence (IC) is defined as [87]:

(A7) $$IC=\frac{1}{N}\sum_{i=1}^{N}1_{\left(y_{i}^{c}<O_{i}^{c}\right)}$$

where:

- $y_{i}^{c}$ represents the model’s confidence for class *c* of the *i*th sample when the entire image is used as input.
- $O_{i}^{c}$ represents the model’s confidence for class *c* of the *i*th sample when only the important regions identified by the explanation map are kept, and the remaining regions are replaced with the image mean.
- $1_{\left(\cdot \right)}$ is an indicator function that returns 1 when the argument is true (i.e., when $O_{i}^{c}>y_{i}^{c}$) and 0 otherwise.

Higher IC values indicate that the explanation successfully identifies decision-critical regions.
